# Imaging in malignant germ cell tumors involving the hypothalamo-neurohypophyseal axis: the evaluation of the posterior pituitary bright spot is essential

**DOI:** 10.1007/s00234-024-03384-1

**Published:** 2024-06-07

**Authors:** Annika Stock, Gabriele Calaminus, Mathilda Weisthoff, Julia Serfling, Torsten Pietsch, Brigitte Bison, Mirko Pham, Monika Warmuth-Metz

**Affiliations:** 1 Department of Neuroradiology, University Hospital Wuerzburg, Josef-Schneider-Strasse 11, D-97080 Wuerzburg, Germany; 2 Department of Pediatric Hematology/Oncology, University Children’s Hospital Bonn, Bonn, Germany; 3 Present Address: Department of Radiology, University Hospital Cologne, Colonge, Germany; 4 Present Address: Department of Diagnostic and Interventional Radiology, University Hospital of Wuerzburg, Wuerzburg, Germany; 5 Institute of Neuropathology, DGNN Brain Tumor Reference Center, University of Bonn Medical Center, Bonn, Germany; 6 Diagnostic and Interventional Neuroradiology, Faculty of Medicine, University of Augsburg, Augsburg, Germany; 7 Neuroradiological Reference Center for the pediatric brain tumor (HIT) studies of the German Society of Pediatric Oncology and Hematology, University Hospital Wuerzburg until 2020; Diagnostic and Interventional Neuroradiology, Faculty of Medicine, University of Augsburg Since 2021, Augsburg, Germany

**Keywords:** Pituitary gland, Posterior pituitary bright spot loss, Germ cell tumor, MRI, Imaging, Child, Pediatric, Diabetes insipidus

## Abstract

**Purpose:**

Malignant intracranial germ cell tumors (GCTs) are rare diseases in Western countries. They arise in midline structures and diagnosis is often delayed. We evaluated imaging characteristics and early tumor signs of suprasellar and bifocal GCT on MRI.

**Methods:**

Patients with the diagnosis of a germinoma or non-germinomatous GCT (NGGCT) who received non-contrast sagittal T1WI on MRI pre-therapy were included. Loss of the posterior pituitary bright spot (PPBS), the expansion and size of the tumor, and the expansion and infiltration of surrounding structures were evaluated. Group comparison for histologies and localizations was performed.

**Results:**

A total of 102 GCT patients (median age at diagnosis 12.3 years, range 4.4–33.8; 57 males; 67 in suprasellar localization) were enrolled in the study. In the suprasellar cohort, NGGCTs (*n* = 20) were noticeably larger than germinomas (*n* = 47; *p* < .001). Each tumor showed involvement of the posterior lobe or pituitary stalk. A PPBS loss (total *n* = 98) was observed for each localization and entity in more than 90% and was related to diabetes insipidus. Osseous infiltration was observed exclusively in suprasellar GCT (significantly more frequent in NGGCT; *p* = .004). Time between the first MRI and therapy start was significantly longer in the suprasellar cohort (*p* = .005), with an even greater delay in germinoma compared to NGGCT (*p* = .002). The longest interval to treatment had circumscribed suprasellar germinomas (median 312 days).

**Conclusion:**

A loss of the PPBS is a hint of tumor origin revealing small tumors in the neurohypophysis. Using this sign in children with diabetes insipidus avoids a delay in diagnosis.

## Introduction

Intracranial malignant germ cell tumors (GCTs) are a rare disease in European countries. There is a slightly higher incidence in boys, with a median age at diagnosis of 11.4 years (for girls, 9.8 years) and a 5-year survival rate of 85% [1]. The World Health Organization (WHO) -classification of central nervous system tumors divides histologically between malignant germinoma and non-germinomatous germ cell tumors (NGGCT) [2]. Where intracranial GCTs originate is not yet clarified. There are two main theories, one that hypothesizes the mismigration of primordial germ cells along the sympathetic trunk in the midline of the body in the human embryo and the other that suggests the transformation of endogenous brain cell progenitors [3–6]. Intracranial GCTs are primarily located in the pineal gland, followed by the suprasellar region [7]. In Western countries, the bifocal localization (suprasellar and pineal tumor proportion) is more frequent than in East Asia [8].

The posterior pituitary gland (neurohypophysis) develops from the hypothalamus and is divided into the intrasellar pars nervosa and the infundibulum (pituitary stalk). The pars nervosa is composed of axonal terminations of neurons and pituicytes. Moreover, it secretes the hormones oxytocin and vasopressin, which can be visualized on T1WI in MRI. Radiologically the preserved posterior pituitary bright spot (PPBS) demonstrates the integrity of the neurohypophysis. GCT patients with tumors involving the hypothalamo-neurohypophyseal axis often present with endocrinopathies, most commonly diabetes insipidus (DI), and visual disturbances [9]. The disturbed secretion of vasopressin can lead to both the clinical manifestation of DI and, on MRI, to a loss of PPBS. That a loss of the PPBS is associated with DI which may indicate a suprasellar GCT has been the object of small suprasellar germinoma cohorts in the past [10–14]. Nevertheless, a delay in diagnosis of suprasellar GCT has been frequently reported [15, 16]. But early tumor diagnosis is important, because the risk of dissemination is increased with prolonged symptom intervals [17].

Therefore, we evaluated cranial MRI scans for the PPBS sign in a large Western European cohort of malignant GCT involving the hypothalamo-neurohypophyseal axis. We hypothesize that the loss of the PPBS is an early radiological tumor maker. We also investigated whether the localization of small tumors provides an indication of the site of origin of GCTs.

## Materials and methods

### Patients

In this retrospective evaluation only GCT patients with given written consent (by patients or parents) to the SIOP-CNS-GCT-96 or SIOP-CNS-GCT II trial were included. The detection of an intact PPBS is based ideally on unenhanced thin sagittal T1WI. Between August 2006 and December 2019 there were 102 bifocal and suprasellar GCT patients with a pre-treatment MRI, including a sagittal non-contrast T1WI, in the database of the National Reference Center for Neuroradiology. The tumor entity was confirmed by histology or tumor markers (alpha-1-fetoprotein > 25ng/mL and/or human chorionic gonadotropin > 50 IU/L) in at least one serum/cerebrospinal fluid compartment [18]. Patient’s symptoms at diagnosis like diabetes insipidus (DI), endocrinological dysfunction or disturbances of visual capacity were documented when available.

### Imaging

The MRI data originated from multiple treating hospitals with differences in MRI scanner manufacturers and field-strength. Two neuroradiologists (M. W.-M., A. S.) separately evaluated the tumor expansion, the presence of the PPBS and affection of surrounding structures. In cases of disagreement consensus reading was performed.

The tumor volume was measured in three orthogonal dimensions (transversal, sagittal and coronal) and was calculated in cm³ using the rotation ellipsoid formula (AxBxC)/2. In bifocal GCTs, the volume was calculated for both tumor portions. The suprasellar tumor growth was rated as: (1) circumscribed, when the tumor grew within the pituitary gland or stalk and expanded to the floor of the third ventricle or optic chiasm; (2) extended, when the tumor grew from the pituitary gland or stalk to the third ventricle with a tumor portion within the third ventricle or compression of diencephalic structures like the mammillary bodies or the mesencephalon.

The diameter of the pituitary stalk was measured in tumors, where the shape of the stalk was nearly preserved. The diameter was measured anterior-posterior at the entrance into the sellar diaphragm and at the level of the optic chiasm. According to current literature, there are no established limits for the thickness of the pituitary stalk in children. Since our cohort mainly consists of children, we have adhered to consensus recommendations, setting the cut-off at 3 mm [19, 20]. Therefore, it was further evaluated how often the diameter did not exceed 3 mm.

**Fig. 1:**
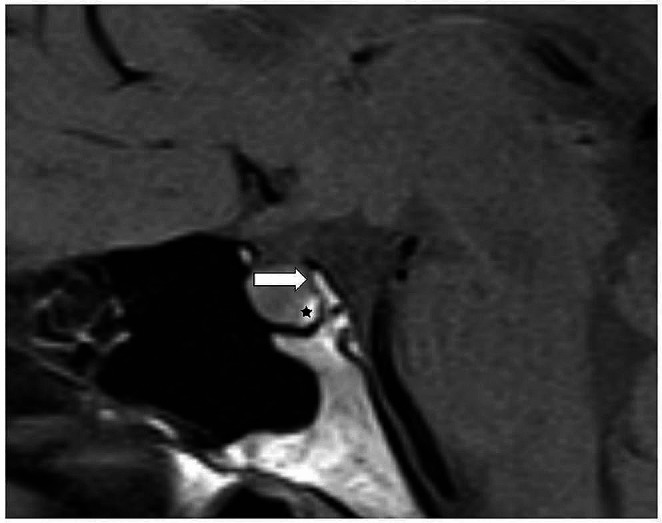
A T1-weighted sagittal image without contrast in a healthy person. The arrow marks the cortical layer of the clivus, which presents as a physiological border between bone marrow fat and the bright spot (*) of the posterior pituitary lobe

The cortical layer of the clivus is adjacent to the posterior pituitary gland and determinable as a dark ribbon in T2WI and T1WI (Fig. 1). When this ribbon was not detectable, osseus destruction was assumed. Furthermore, the cases were evaluated for edema along the optic tracts as a sign of tract compression.

Intracranial dissemination was assessed as laminar (M2a) or nodular (M2b) [21]. The cases in which the T1WI post-contrast did not cover the entire neurocranium were excluded for the evaluation of leptomeningeal dissemination.

### Statistical analysis

Medians with ranges are given for metric data (age, measurements, and time interval). The time between the first MRI performed due to specific symptoms suspicious of a GCT and the therapy start or surgery was calculated in days. Categorial variables are indicated in absolute or relative frequencies. To correlate clinical and tumor information with imaging findings, each encoded in categorial variables, Chi-square or Fisher exact test was performed. Group comparison between the medians of categorical variables was performed with the Mann-Whitney-U-test. Even though this was an explorative analysis, *p* values < 0.05 were interpreted as significant.

## Results

### Patients

Inclusion criteria were fulfilled in 102 GCT patients, 67 were in suprasellar and 35 in bifocal localization (epidemiology, clinical and imaging data in Table 1). Nearly two-third of bifocal and suprasellar GCT were diagnosed as germinomas (bifocal 74.3%, suprasellar 70.1%). Median age at diagnosis was 12.3 years. Patients of the suprasellar cohort were significantly younger (*p =* .018) and the bifocal cohort contained more male patients (*p =* .007).

**Table 1:** Epidemiologic data and imaging analysis

	total cohort	suprasellar GCT				bifocal GCT				*p*-value
		total	germinoma	NGGCT	*p*	total	germinoma	NGGCT	*p*	
*n*	102	67	47 (70.1%)	20 (29.9%)		35	26 (74.3%)	9 (25.7%)		
age at MR diagnosis (median and range in years)	12.3 (4.4–33.8)	11.7 (4.4–33.8)	12.2 (5.1–33.8)	10.2 (4.4–21.8)	0.07	13.3 (7.8–26.1)	13.4 (9.9–22.4)	13 (7.8–26.1)	0.64	**0.018**
gender					0.45					**0.007**
- male - female	57 (55.9%) 45 (44.1%)	31 (46.3%) 36 (53.7%)	23 (48.9%) 24 (51.1%)	8 (40%) 12 (60%)		26 (74.3%) 9 (25.7%)	21 (80.8%) 5 (19.2%)	5 (55.6%) 4 (44.4%)	0.19	
time to therapy (median with range in days)	18.5 (0-1102)	24 (0-1102)	56 (0-1102)	8 (0-1046)	**0.002**	9 (1-204)	10 (1-204)	9 (1–98)	0.88	**0.005**
DI total (*n* = 45)	45	31 (68.9%)	21 (67.7%)	10 (32.3%)	n. e.	14 (31.1%)	8 (57.1%)	6 (42.9%)	n. e.	n. e.
symptoms (*n* = 61)					n. e.				n. e.	n. e.
- only DI - DI + endoDys - DI + endoDys + visus - DI + visus - endoDys - endoDys + visus - visus only - others*	13 (21.3%) 24 (39.3%) 5 (8.2%) 2 (3.3%) 2 (3.3%) 4 (6.6%) 4 (6.6%) 7 (11.5%)	4 (10%) 21 (52.5%) 5 (12.5%) 1 (2.5%) 2 (5%) 4 (10%) 3 (7.5%) 0	2 (7.4%) 17 (63%) 2 (7.4%) 0 2 (7.4%) 2 (7.4%) 2 (7.4%) 0	2 (15.4%) 4 (30.8%) 3 (23.1%) 1 (7.7%) 0 2 (15.4%) 1 (7.7%) 0		9 (42.9%) 3 (14.3%) 0 1 (4.8%) 0 0 1 (4.8%) 7 (33.3%)	5 (35.7%) 2 (14.3%) 0 0 1 (7.1%) 0 1 (7.1%) 5 (35.7%)	4 (57.1%) 1 (14.3%) 0 0 0 0 0 2 (28.6%)		
tumor size (cm³)										
- sellar - pineal	1.3 (0.05–36.2) 1.7 (0.4–16.4)	1.8 (0.1–36.2)	0.9 (0.1–36.2)	8.6 (0.1–31.9)	**< 0.001**	0.8 (0.05–21.8) 1.7 (0.4–16.4)	0.8 (0.05–17.6) 1.7 (0.4–16.4)	0.7 (0.4–21.8) 1.6 (0.4–4.8)	0.6 0.79	0.2
tumor expansion										
- circumscribed - expanded	39 (38.2%) 63 (61.8%)	28 (41.8%) 39 (58.2%)	25 (53.2%) 22 (46.8%)	3 (15%) 17 (85%)	**0.004**	11 (31.4%) 24 (68.6%)	8 (30.8%) 18 (69.2%)	3 (33.3%) 6 (66.7%)	1	0.31
tumor involved structures										
- PL - PS - neither PL nor PS - 3rd ventricle floor - optic chiasm - 3rd ventricle - MB - mesencephalon - lateral ventricles - brain parenchyma	89 (87.3%) 100 (98%) 0 89 (87.3%) 92 (90.2%) 62 (60.8%) 37 (36.3%) 14 (13.7%) 4 (3.9%) 4 (3.9%)	63 (94%) 65 (97%) 0 55 (82.1%) 58 (86.6%) 38 (56.7%) 26 (38.8%) 11 (16.4%) 1 (1.5%) 3 (4.5%)	44 (93.6%) 46 (97.9%) 0 37 (78.7%) 39 (83%) 21 (44.7%) 14 (29.8%) 5 (10.6%) 1 (2.1%) 3 (6.4%)	19 (95%) 19 (95%) 0 18 (90%) 19 (95%) 17 (85%) 12 (60%) 6 (30%) 0 0	1 0.51 0.49 0.26 **0.002** **0.02** 0.07 1 0.55	26 (74.3%) 35 (100%) 0 34 (97.1%) 34 (97.1%) 24 (68.6%) 11 (31.4%) 3 (8.6%) 3 (8.6%) 1 (2.9%)	17 (65.4%) 26 (100%) 0 25 (96.2%) 25 (96.2%) 18 (69.2%) 9 (34.6%) 2 (7.7%) 1 (3.8%) 1 (3.8%)	9 (100%) 9 (100%) 0 9 (100%) 9 (100%) 6 (66.7%) 2 (22.2%) 1 (11.1%) 2 (22.2%) 0	**0.07** n.d. n.d. 1 1 1 0.69 1 0.16 1	**0.01** 0.55 n.d. **0.032** 0.16 0.24 0.46 0.37 0.12 1
diameter of the pituitary stalk (median and range in mm)**										
- superior (*n* = 61) - inferior (*n* = 64)	6.1 (2.2–12.9) 3.5 (0.6–11.4)	5.4 (2.2–12.9) 3.5 (1.2–11.4)	5.6 (2.2–12.9) 3.5 (1.2–10.9)	5.2 (2.7–8.3) 5.7 (1.5–11.4)	0.75 0.2	6.6 (3.9–11.3) 3 (0.6–5.4)	6.9 (3.9–11.3) 2.3 (0.6–5.3)	6.6 (5-8.2) 3.9 (3.5–5.4)	0.92 **0.036**	0.09 0.06
inferior diameter:					0.38				**0.014**	0.33
≤ 3 mm > 3 mm	27 (42.2%) 37 (57.8%)	14/40 (35%) 26/40 (65%)	13/34 (38.2%) 21/34 (61.8%)	1/6 (16.7%) 5/6 (83.3%)		12/24 (50%) 12/24 (50%)	12/18 (66.7%) 6/18 (33.3%)	0/6 6/6 (100%)		
loss of the bright spot	98/102 (96.1%)	64/67(95.5%)	46 (97.8%)	18 (90%)	0.210	34/35 (97.1%)	25/26 (96.2%)	9/9 (100%)	1	1
optic tract edema^1^	36/101 (35.6%)	22/66 (33.3%)	12/46 (26.1%)	10/20 (50%)	0.06	14/35 (40%)	8/26 (30.8%)	6/9 (66.7%)	0.11	0.51
osseus infiltration^2^	18/97 (18.6%)	18/65 (27.7%)	8/46 (17.4%)	10/19 (52.6%)	**0.004**	0	0	0		**< 0.001**
dissemination at diagnosis^3^	17/90 (18.9%)	7/56 (12.5%)	7/39 (17.9%)	0/17	0.09	10/34 (29.4%)	9/26 (34.6%)	1/8 (12.5%)	0.39	**0.047**

Table 1 provides an overview of patient and imaging data, including comparisons between the suprasellar and bifocal groups, and additionally between germinoma and non-germinomatous germ-cell tumors per localization group. Clinical data were available for 61 patients, of which 45 reported DI either alone or combined with endocrine or visual disturbances. Abbreviations: DI = diabetes insipidus; MB = mammillary bodies; n. e. = not evaluated for dependencies, because of the lack of information’s; n.d.= not determinable; NGGCT = non-germinomatous germ cell tumor; PL = posterior lobe of the pituitary gland; PS = pituitary stalk; endoDys = endocrine dysfunction;

* Headaches, vomiting, appetite loss, fatigue, aggressivity and changes in behavior

** the inferior stalk was measurable in 64 patients (40 germinoma, 24 non-germinomatous germ-cell tumors)

^1^ Not assessable in one patient

^2^ Not assessable in three patients due to image quality

^3^ Not assessable in 13 patients

Information about patients’ symptoms were available in 61. In total, 45 patients showed symptoms of DI at diagnosis but only in 13 patients this was a single symptom. The most common clinical presentation in the suprasellar cohort was DI (52.5%) in combination with at least one disturbance of the ventral pituitary lobe, suggesting endocrine dysfunction. In contrast, the bifocal cohort presented most often with DI alone (42.9%) or other symptoms like headaches, vomiting, appetite loss, fatigue and behavioral changes (33.3%). Impairment of the visual system was present in 15 patients but in 11/15 combined with other symptoms.

### Imaging

Tumor size was at a median of 1.8 cm^3^ in suprasellar GCT and 0.8 cm^3^ in bifocal GCT. In the suprasellar cohort, germinomas were significantly smaller than NGGCTs (*p* < .001). But in the bifocal cohort, the entities showed comparable sizes of the suprasellar tumor portion. The tumor portion within the pineal gland was larger with a median of 1.7 cm3 and germinoma and NGGCT did not differed significantly.

The suprasellar tumor portion in both localizations most often expanded (61.8%) into the third ventricle or compressed diencephalic structures. Within in the suprasellar cohort, NGGCT were significantly more often expanded than circumscribed compared to germinoma (85% vs. 46.8%, *p = .*004). In this aspect, NGGCT and germinoma of the bifocal cohort did not differ significantly. Thirteen circumscribed tumors (12 in suprasellar localization) did not involve the floor of the third ventricle but 6 of them affected the optic chiasm.

**Fig. 2:**
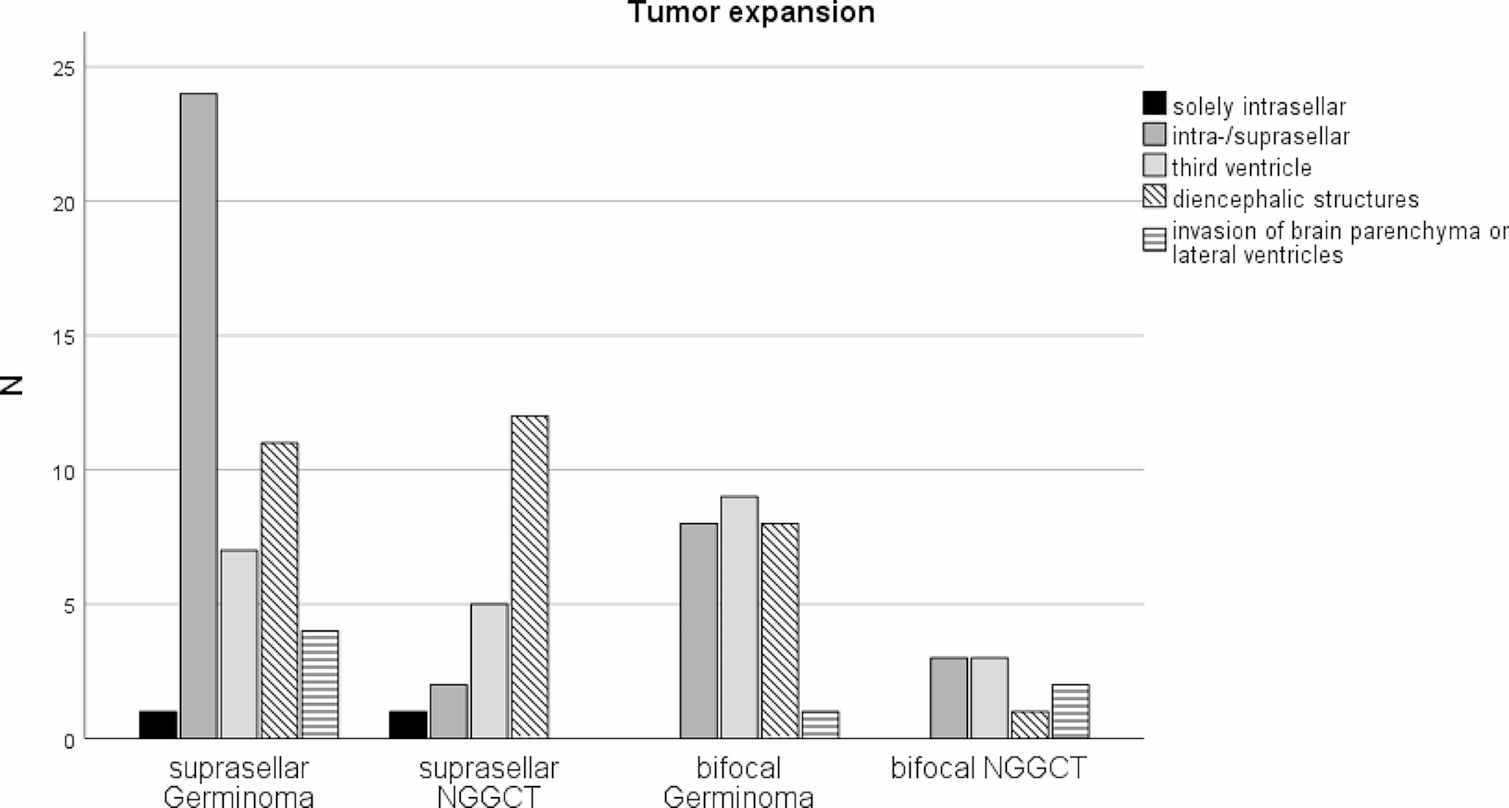
The figure illustrates the tumor expansion observed in suprasellar tumors, delineating the proportion of both suprasellar and bifocal germinomas, as well as non-germinomatous germ-cell tumors (NGGCT). Tumors solely affecting the posterior lobe of the pituitary gland were infrequent (*n* = 2) and were exclusively identified in cases of suprasellar GCT. In instances categorized as ‘intra-/suprasellar,’ tumors extended towards the optic chiasm or the floor of the third ventricle. Compression of diencephalic structures occurred when the mammillary bodies or the mesencephalon were affected. Extensive expansions into the lateral ventricles or invasion of brain parenchyma were relatively uncommon in suprasellar GCT compared to bifocal cases

Each tumor involved the pituitary gland or the stalk. No involvement of the posterior pituitary gland was found in 13 tumors (4 suprasellar, 9 bifocal). In these cases, tumor was present in the pituitary stalk. Both structures were infiltrated simultaneously in 87. The posterior lobe was less often involved in bifocal germinoma (65.4%) than in NGGCT (100%; *p = .*07), suprasellar germinoma (93.6%) and suprasellar NGGCT (95%; group comparison suprasellar vs. bifocal: *p = .*01). In the suprasellar cohort, growth into the third ventricle (*p* = .002) and affection of the mammillary bodies (*p* = .02) was significantly more frequent in NGGCT than in germinoma. The tumor expansion is illustrated in Fig. 2.

**Fig. 3:**
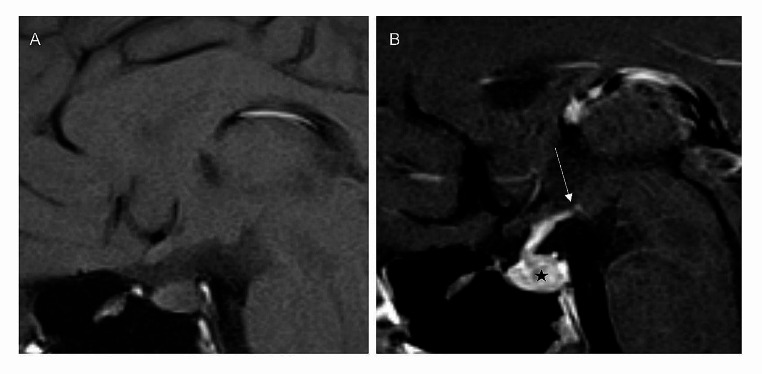
The first MRI of a patient with diabetes insipidus. (**A**) Sagittal non-contrast T1-weighted image shows the absence of the posterior pituitary bright spot. (**B**) Sagittal contrast-enhanced T1-weighted image with 2 mm slice thickness shows a small germinoma (black star) in the posterior pituitary lobe and inferior pituitary stalk, with a subtle thickening of the floor of the third ventricle. Please note that the contrast enhancement extends to the mammillary bodies (arrow), indicating a pathological condition. The diagnosis was confirmed by surgery 19 months later

**Fig. 4:**
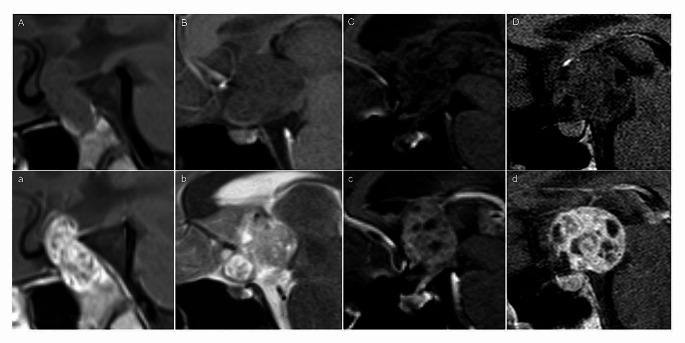
The upper row demonstrates the four cases with a preserved posterior pituitary bright spot on sagittal T1-weighted images without contrast. The cases are named as in Table 2. Especially case D shows an irregular bright spot. The lower row shows the tumor appearance in post-contrast T1-weighted images (**a**, **c** + **d**) and T2-weighted image (**b**; in that case, no post-contrast sagittal T1-weighted image was available)

All patients with clinical information of DI showed no PPBS (one example in Fig. 3). Of the four cases with a PPBS, only one patient had available clinical information and no DI was reported. That patient presented endocrine dysfunction and visual disturbance. The PPBS was present in 4/102 cases (4%; Table 2; Fig. 4) without differences between histologies or localizations. All four cases needed a consensus between the readers, because there was only a slight but not regular appearing hyperintensity of the posterior pituitary lobe. Two cases of expanded tumors showed a PPBS while an intrasellar tumor portion was present.

**Table 2:** Cases without loss of the posterior pituitary bright spot

case	localization	diagnosis	gender	DI	age at diagnosis (years)	suprasellar tumor size (cm³)	tumor expansion	tumor proportion intrasellar	thickness of the inferior pituitary stalk
A	suprasellar	NGGCT	female	no	8	1.8	expanded	yes	11.4 mm
B	suprasellar	NGGCT	male	NA	14	5.7	expanded	no	non measurable
C	bifocal	germinoma, metastasized	male	NA	18	7.2	expanded	no	1.1 mm
D	suprasellar	germinoma	male	NA	18	11	expanded	yes	non measurable

Table 2 shows the four cases with a present bright spot of the posterior pituitary gland according to the arrangement in Fig. 4. In case B and D the shape of the pituitary stalk was not preserved. Abbreviations: NA = no information available; NGGCT = non-germinomatous germ-cell tumor

**Table 3:** Tumor data and characteristics by expansion

	suprasellar, circumscribed *n* = 28	suprasellar, expanded *n* = 39	*p*-value	bifocal, circumscribed *n* = 11	bifocal, expanded *n* = 24	*p*-value
size of the suprasellar tumor portion (cm^3^, median and range)	0.44 (0.08–16.73)	7 (0.23–36.24)	**< 0.001**	0.39 (0.05–0.72)	3.1 (0.16–21.84)	**< 0.001**
time between first abnormal MRI and therapy start (days)			**< 0.001**			0.52
• median • IQR	312 (0-1102) 108; 658	9 (0-619) 5; 24		12 (1-204) 5; 44	8 (1-182) 3; 23	
bright spot present	0	3 (7.7%)	0.26	0	1 (4.2%)	1
posterior lobe involvement	27 (96.4%)	36 (92.3%)	0.64	7 (63.6%)	19 (79.2%)	0.42
pituitary stalk involvement	26 (92.9%)	39 (100%)	0.17	11 (100%)	24 (100%)	n.d.
size of the inferior stalk	*n* = 25	*n* = 15		*n* = 11	*n* = 13	
• median (range) • IQR	3.1 (1.3–7.6) 2.1; 4.6	5.5 (1.2–11.4) 3.5; 7.4	**0.017**	2.7 (0.6–5.4) 1.8; 3.9	3.6 (1.1–5.3) 1.7; 4.7	0.49

Table 3 divides between expanded GCTs and circumscribed ones. Circumscribed was defined as an expansion until the floor of the ventricle or reaching the optic chiasm. Abbreviations: n.d.= not determinable; IQR = interquartile range, given are percentile 25 and 75

The inferior pituitary stalk diameter was measurable in 64 patients with a median of 3.5 mm (range 0.6–11.4). A diameter ≤ 3 mm was observed in 15/40 suprasellar (14 germinoma) and 12/24 bifocal (all germinoma) GCT. The diameter was substantially smaller in bifocal germinoma compared to NGGCT (*p* = .014). The superior pituitary stalk diameter was measurable in 61 of the 64 patients (median 6.1 mm).

Optic tract edema was non-significantly more present in NGGCT (suprasellar 50%, bifocal 66.7%) than in germinoma in the suprasellar (26.1%, *p = .*06) and in the bifocal cohort (30.8%; *p =* .11). Of the 15 patients with visual disturbances, each tumor showed contact to or compression of the optic chiasm while optic tract edema was rather rare (*n* = 4).

Destruction of the cortical layer of the clivus, assuming osseous infiltration, was exclusively present in suprasellar GCT (*n* = 18) and significantly more frequent in NGGCT than in germinoma (*p =* .004).

Contrast T1WI covering the whole neurocranium was available in 90 patients and demonstrated intracranial leptomeningeal dissemination at diagnosis in 17 patients (more common in the bifocal cohort; *p = .*047). No suprasellar NGGCT showed leptomeningeal dissemination and bifocal NGGCTs showed less leptomeningeal dissemination than germinomas, but these results were not significant (suprasellar *p =* .09, bifocal *p = .*39).

### Diagnosis and Delay

For the suprasellar cohort, 24 clinical reports offered information about the duration of symptoms before the first MRI was performed. In nine patients, there was no relevant delay until the first MRI. Five patients underwent a first MRI with a latency of a few months (range between 3 and 7 months) and in further nine patients, the symptoms persisted for more than one year (up to 4 years in a patient with growth restriction and 3 years in one patient with diabetes insipidus). Only five clinical reports were available in the bifocal cohort. Here symptoms occurred rather acute (two patients with DI and another one with nausea for four weeks) or persisted for a maximum of six months (two patients with weight loss).

**Fig. 5:**
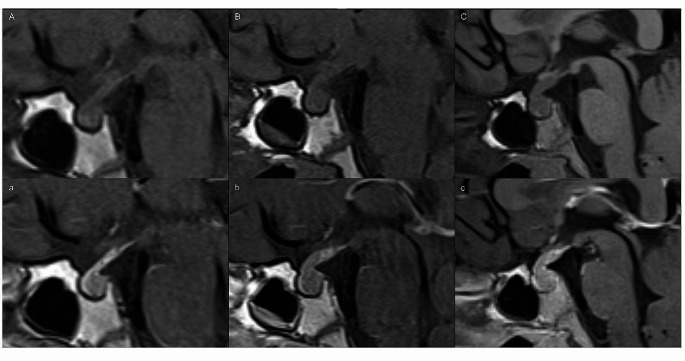
A case of a prolonged diagnosis. The patient experienced polydipsia and headaches for several months. The initial MRI examination revealed a loss of the posterior pituitary bright spot on a sagittal T1WI without contrast (**A**), and after gadolinium application (**a**), a tumor was found in the posterior pituitary gland.  T1-weighted images in (**B**, without contrast) and (**b**, with contrast) showed tumor growth and progressive pituitary stalk thickening 12 months later. Preoperative planning (**C**, **c**) did not occur until 20 months after the first MRI

Time between the first MRI being assessed as abnormal and the first treatment accounted for a median of 18 days. Treatment was initiated significantly more rapid in the bifocal cohort with a median of 10 days but 24 days in the suprasellar cohort, *p =* .005, without differences between the histological groups. Within the suprasellar cohort therapy started significantly later in germinoma patients than in patients with a NGGCT (*p =* .002). Furthermore, in suprasellar circumscribed tumors the longest intervals were found with a median of 312 days (Table 3, one example is given in Fig. 5). In contrast, suprasellar expanded tumors were treated after a median of 9, and bifocal circumscribed tumors after a median of 12 days.

## Discussion

Within our large multi-center cohort, we found a nearly 100% rate of PPBS loss in germinoma and NGGCT of the hypothalamo-neurohypophyseal axis. This finding is important in pediatric imaging, because it serves as a rather reliable diagnostic criterion for early tumor detection in malignant GCTs and has the potential to reduce delays in diagnosis.

GCTs are the most prevalent suprasellar pathology in pediatric patients with DI, particularly in the Asian population [22]. In the general population, an absence of the PPBS is observed in 4.1% of individuals without endocrinopathies, with a correlation to increasing age [23]. In a small cohort of 17 GCTs, absence of the posterior pituitary bright sport was reported in 82% [10]. We observed a loss of the PPBS in 96.1% in the total cohort and in the cases with available clinical information regarding an existing DI, each MRI revealed a PPBS loss.

The occurrence of a loss of the PPBS in other sellar tumors, such as adenomas, is uncommon, accounting for a mere of 20% [24]. Furthermore, adenoma exhibit an expansion of the adenohypophysis, whereas microadenomas are distinguished by sparse areas of contrast enhancement. The distinction between small GCTs and Langerhans cell histiocytosis may pose a greater challenge on MRI. However, the evaluation of alpha-1-fetoprotein and beta human chorionic gonadotropin can serve as an additional diagnostic tool to ascertain NGGCTs and in non-secreting tumors skeletal evaluation may provide the diagnosis of a sellar lesion. Idiopathic central diabetes determined by inflammatory/autoimmune causes is noted as one important differential diagnosis especially in cases with pituitary stalk thickening [25]. Since pituitary stalk thickening in inflammatory diseases should regress within the first six months, close clinical follow-up is necessary for a differentiation from small GCTs [26, 27]. However, it must be noted that pure germinomas are often associated with immune cell infiltration, and therefore, treatment with corticosteroids could lead to an apparent reduction in tumor size in the short-term follow-up [28].

Histologically, intracranial GCT do not differ from their extracranial counterparts. Furthermore, extra- and intracranial GCT most often occur within the midline. It is hypothesized that GCTs develop from non-eliminated mismigrated primordial germ cells that migrate along the sympathetic trunk can reach the midline of the brain [3]. Teilum hypothesized that extragonadal GCT, germinoma and NGGCT, originate from stray primordial germ cells [29]. But germinoma are more sensitive to radiotherapy and chemotherapy which exhibits a better prognosis compared to NGGCT. This may be explained by their rapidly progressive, undifferentiated cells leading to the assumption that NGGCT differentiate from different cells of origin. Therefore, it is postulated that the primordial germ cells theory rather explains the evolution of germinomas, but for NGGCT the pluripotent stem cell hypothesis is more plausible [30]. Recently, it was suggested that the transformation from primordial germ cells to transformed embryonic stem cells is the most logical mechanism for all intracranial GCTs [31]. Both theories do not explain the midline location sufficiently, the authors assumed lateral ventricle location of GCT because neural stem cells are present in the subependymal zone. We only evaluated midline location of GCT and each tumor in our study involved the neurohypophysis, where hypothalamic neurons terminate. However, this does not explain the location as well. The fact that small tumors were almost exclusively located inferior to the optic chiasm may be a further hint of tumor origin. In these cases, the diagnostic value of the loss of PBBS is profound. Already Fujisawa et al. [32] postulated on a basis of seven patients that germinoma of the hypothalamo-neurohypophyseal axis arise from the neurohypophysis and a theory by Tan [4] was that anterior midline structures are highly active in hormone production and concentration in factors like GnRH, potentially playing a role in tumorigenesis. Ultimately, radiology cannot provide a definitive explanation for the origin of GCTS; however, we aspire that our findings will aid in the advancement of theories. Recently a novel MRI classification for intracranial GCT, compromising bifocal and basal ganglia manifestations, was introduced [33]. Within this report no GCT was localized only intrasellar. Therefore, they hypothesized that suprasellar GCT arise from the tuber cinereum and median eminence and infiltrate the stalk. We found 13 GCTs in the neurohypophysis not involving the third ventricle floor and therefore, we cannot support the results. One explanation for these different results may be that Esfahani et al. [33] evaluated only post-contrast T1WI.

In general, each tumor in our cohort involved the posterior pituitary gland or the stalk and most tumors (61.8%) showed an expanded growth with compression of diencephalic structures. While suprasellar and bifocal location did not differ, we found that suprasellar NGGCT were significantly more often (85%) expanded compared to suprasellar germinoma (46.8%). In bifocal GCT the posterior pituitary was significantly less often involved than in the suprasellar cohort, but this may result from the lower incidence of bifocal germinoma. The pituitary stalk was involved in nearly 100% in both location groups and was independent from histology. In the work by Zhang et al., the posterior lobe was less often infiltrated than the stalk in a bifocal GCT cohort [34]. The involvement of the suprasellar region was deemed as a metastatic seeding from a primary pineal GCT and rated as “false” bifocal location. False GCT were differentiated from true GCT by their shape. Interestingly, the tumors rated being “false” bifocal, showed no involvement of the posterior lobe. In four cases we found a present PPBS, but only one in a bifocal metastasized germinoma. Finally, the signal intensity of the PPBS in all four cases was lower than expected or not well delimitable to the clivus and accordingly, consensus reading was necessary (of a total of 11 consensus readings).

It may be challenging to distinguish bone marrow fat from the PPBS on MRI. In GCTs, the cortical layer of the clivus is not a reliable tool for spatial delineation, because, as we observed in suprasellar GCTs, it can be destroyed or infiltrated by the tumor. As a consequence, we propose that each patient with endocrinopathy should be evaluated by a non-contrast sagittal T1WI with fat-suppression for a better discrimination.

Kilday et al. [10] argued that a present PPBS in germinoma indicates residual pituitary function. Unfortunately, we do not have sufficient data on DI and serum sodium values in our patients to support this hypothesis. In our cohort, each patient with DI or polydipsia had no PPBS at diagnosis.

Consensus recommendations set a cut off of the diameter of the inferior pituitary stalk of 3 mm [19, 20]. Our study revealed that 27 out of the 64 (42.2%) measurable inferior stalks had a diameter within that range indicating that this is not a reliable indicator for early tumor detection. Zhang et al. [34] assumed that bifocal intracranial germinoma are “false”, if the inferior pituitary stalk is measuring less than 3.0 mm or the bright sport sign is absent. We observed only one bifocal germinoma patient with a PPBS and an inferior stalk measuring 1.1 mm. In addition, this patient showed true leptomeningeal dissemination and therefore the “false” bifocal theory is quite possible.

There was, however, no noticeable difference in the diameter of the inferior stalk between suprasellar and bifocal GCT in our study. Of note, the diameter was substantially smaller in bifocal germinoma compared to NGGCT (*p* = .014) and in 40% within the range of the healthy population.

Even if the spectrum of causes for a PPBS loss is wide, each differential diagnosis needs rapid clarification. In pediatric GCT patients, a delay in diagnosis has been reported in 83% with a median time interval between symptom onset and diagnosis of 25 months [16]. It is known that prolonged symptom intervals in GCT patients are associated with higher risk of developing a leptomeningeal dissemination [17]. Furthermore, poorer outcomes have been reported for germinoma patients treated after a delay [15]. The PPBS loss is only systematically reported in suprasellar germinoma [10]. With our work, we are the first to report this sign as common also in NGGCT. Our results led us to conclude that the tumor could be overlooked on the first MRI. For early tumor detection, especially small GCTs require a sufficient marker. The earlier treatment start in the bifocal cohort was expectable, but the maximum interval to treatment of 204 days in bifocal germinoma demonstrates the difficulty in smaller tumors as well. Regarding bifocal GCT, it is important to note the recently published consensus recommendation on GCT in this context. When central diabetes insipidus is present with the absence of the PPBS on MRI, and the pituitary stalk or median eminence is not thickened, patients with pineal tumors and negative serum and cerebrospinal fluid α-fetoprotein and human chorionic gonadotropin markers should be considered as having sufficient evidence of bifocal germinoma [20].

In our cohort, there was a delay in the time interval between MRI and treatment start, which was significantly longer in the suprasellar cohort (median 9 days in the bifocal cohort versus median 24 days in the suprasellar cohort). Moreover, the time interval was more prolonged in suprasellar germinoma compared to NGGCT (median 54 days versus 8 days) and in suprasellar circumscribed tumors when compared to expanded tumors (median 312 versus 9 days). We believe that this is caused by the significantly smaller tumor size that we have observed. A mono-institutional study determined the time interval between symptoms onset and diagnosis by surgery or biopsy exactly and reported a median interval of 25 months in sellar germinoma [16]. Because our study was retrospective with patients from multiple hospitals, there was a lack of clinical information. We were able to determine the time interval between the first MRI and the commencement of treatment or surgery. Hence, the duration of the delay is less than 25 months. Nonetheless, it is imperative to assume that the genuine interval based on clinical symptoms is significantly longer, as evidenced by the individual reports with available clinical data in our suprasellar cohort. Clinical reports of the bifocal group were rare (*n* = 5) and the symptom interval with up to six months less prolonged compared to the suprasellar cohort.

## Conclusion

The diagnosis of intracranial germ cell tumors is based on clinical symptoms, measurement of tumor markers (α-fetoprotein and human chorionic gonadotropin) in serum and cerebrospinal fluid, cranial and spinal imaging and cerebrospinal fluid cytology. On imaging, early detection of a PPBS loss is crucial in patients with diabetes insipidus since it is a reliable early sign in GCTs. Furthermore, fat-suppressed imaging might be superior, because it allows a definite distinction from adjacent fat. Each tumor involved the posterior pituitary lobe or stalk. Radiologically GCTs seem to originate from the neurohypophysis but histopathological evidence is missing. The radiologists have a significant objective in reducing the delay in diagnosis by conducting the technically best evaluation. In cases of small suprasellar tumors presenting only with a loss of the PPBS, with or without thickening of the pituitary stalk and without tumor markers, short-term MRI follow-up can be performed. However, to prevent metastasis, a biopsy must ultimately be performed as soon as safely feasible, especially if the findings are not regressive.
